# Principles and Strategies for Interest‐Holder Engagement in Health Guideline Development

**DOI:** 10.1111/jep.70375

**Published:** 2026-02-18

**Authors:** Olivia Magwood, Caitlin Shyng, Lyubov Lytvtyn, Joanne Khabsa, Alex Young‐Soo Lee, Jennifer Petkovic, Vivian Welch, Kevin Pottie, Peter Tugwell

**Affiliations:** ^1^ Interdisciplinary School of Health Sciences, Faculty of Health Science University of Ottawa & Bruyère Health Research Institute Ottawa Ontario Canada; ^2^ Faculty of Science University of Ottawa Ottawa Ontario Canada; ^3^ Department of Health Research, Evidence, and Impact McMaster University Hamilton Ontario Canada; ^4^ Clinical Research Institute American University of Beirut Beirut Lebanon; ^5^ Faculty of Medicine University of Ottawa Ottawa Ontario Canada; ^6^ Faculty of Medicine University of Ottawa & Bruyère Health Research Institute Ottawa Ontario Canada; ^7^ School of Epidemiology and Public Health, Faculty of Medicine University of Ottawa & Bruyère Health Research Institute Ottawa Ontario Canada; ^8^ Faculty of Medicine Dalhousie University, Halifax, Nova Scotia, Canada & Bruyère Health Research Institute Ottawa Ontario Canada; ^9^ Faculty of Medicine & School of Epidemiology and Public Health, University of Ottawa & Clinical Epidemiology Program, Ottawa Hospital Research Institute, & Bruyère Health Research Institute Ottawa Ontario Canada

**Keywords:** clinical practice guidelines, guideline development, interest‐holders, patient and public involvement, stakeholder engagement

## Abstract

**Rationale:**

Guidelines are statements or recommendations that help interest‐holders make decisions about clinical care or health policy. Engaging a wide range of interest‐holders, such as patients, providers, policymakers and members of the public in the guideline development process can help ensure that guidelines are fit‐for‐purpose. The MuSE Consortium is developing guidance for the engagement of interest‐holders throughout the guideline development process in the form of an extension of the Guidelines International Network (GIN)‐McMaster Guideline Development Checklist (GDC).

**Aims and Objectives:**

The objective of this study is to identify principles and strategies to promote interest‐holder engagement across the guideline development enterprise.

**Methods:**

We conducted interviews with 43 individuals from 10 different interest‐holder groups and 15 different countries. We used a framework analysis to thematically analyze findings and identify principles and strategies relevant for engaged guideline development.

**Results:**

Guideline development initiatives may better engage interest‐holders by shifting from vertical to horizontal power structures, emphasizing collaboration and shared decision‐making. We identified a need for epistemic justice, promoting fairness and equality in knowledge production and validation, particularly for patients and members of the public. Representation is a crucial key issue, necessitating diverse perspectives in guideline development groups. Strategies include involving interest‐holders early and meaningfully in the guideline development process through governance, setting clear expectations and timelines, and providing fair compensation. Dissemination activities should extend beyond academic publications, empowering all interest‐holders to contribute to activities such as presentations, educational sessions, or social media campaigns. While engagement is desirable, limitations may arise in emergency contexts or resource‐constrained settings.

**Conclusions:**

Guideline developers may need to make pragmatic decisions as to who they engage in guideline development and how. Capacity strengthening in low‐ and middle‐income countries may help address current disparities in engagement in guideline development. Future research should explore issues around representativeness of interest‐holders.

## 1 Introduction

Guidelines are systematically developed statements to assist decision‐makers such as healthcare providers, patients and policymakers who make decisions about clinical care, public health and health systems [1]. These statements, termed ‘recommendations’, are typically developed through rigorous evidence synthesis and assessments of the benefits and harms of health interventions [1]. Since these recommendations about health and treatment services eventually affect people's lives, engagement in health guideline and policy development is considered a component of people's right to health [2, 3]. Indeed, guideline development organizations such as the World Health Organization (WHO), National Institute for Health and Care Excellence (NICE) and the Guidelines International Network (GIN) have recognized the important role of engaging a range of interest‐holders in the guideline development process [4, 5, 6]. “Interest‐holders” (previously known as “stakeholders” [7];) are individuals and groups with legitimate interests that should be considered in the context of health research. These interests draw their legitimacy from the fact that these people are responsible for or affected by health‐ and healthcare‐related decisions, especially those informed by research evidence [7, 8]. The MuSE Consortium, an international group of over 160 individuals interested in engagement in research and guidelines, has identified 10 types of interest‐holders that are relevant to the context of health guideline development. These include patients and caregivers, payers of health research, purchasers of health services, peer reviewed journal editors, policymakers, principal investigators, product makers, program managers, providers of care, and members of the public [9].

A rigorous guideline development process follows a set of principles including multidisciplinary collaboration and a standardized approach for synthesizing evidence and moving from evidence to recommendations [10, 11]. There are manuals and checklists for guideline development such as the Guidelines International Network (GIN)‐McMaster Guideline Development Checklist (GDC) [12], which is a comprehensive checklist of items that guideline developers could consider for all stages of the guideline enterprise, from the planning and formulation of recommendations to their implementation, evaluation and updating. However, there is a lack of guidance on which interest‐holders should be engaged in guideline development, when during the process they should be engaged, and what principles of engagement should be followed for this engagement to be meaningful. The MuSE Consortium has addressed this gap by developing guidance for engagement of interest‐holders throughout the guideline development process in the form of an extension for engagement of the GIN‐McMaster GDC [13].

Recent work completed by the MuSE Consortium identified gaps in published evidence on the best ways to engage with interest‐holders in health guideline development [14, 15]. Most of the identified evidence was focused on patient and public engagement, with little literature available for other groups such as policymakers, program managers, purchasers, peer review journal editors, product makers, and payers of research. Additionally, there was a clear lack of evidence related to the engagement of people who experience health inequities and/or are from low‐income settings [14]. Similarly, a review by [16]. summarized the available evidence on principles, values, frameworks, and strategies underpinning patient and public engagement in guideline development, but not broader interest‐holder engagement. Furthermore, while they did identify some broad themes such as representation, transparency, relevance, equity, fairness, and reconciling different types of knowledge, the frameworks often failed to explicitly link these principles with the guideline development stages through actionable engagement strategies.

To complement the guidance from the MuSE Consortium on engagement in health guideline development and to promote its operationalization, it is critical to identify principles and strategies for engaging interest‐holders in guideline development that are acceptable to them. This article addresses this need by aiming to identify principles and strategies to promote engagement in the development of health guidelines from the perspective of a broad range of interest‐holders.

## 2 Methods

To inform guidance for the engagement of interest‐holders in health guideline development, the MuSE Consortium conducted a series of reviews, an online survey, key informant interviews and a consensus process. The methods of the project have been reported in the project protocol [17] and guidance paper [13]. This paper reports on findings from the key informant interviews addressing the following research question: What are interest‐holders' perspectives regarding principles and strategies to promote their engagement in the development of health guidelines?

### 2.1 Research Team and Reflexivity

The MuSE Consortium is coordinated by a core team who manage the daily tasks of the group, secure and manage funding for project activities, and communicate frequently with all 160 + members of the MuSE Consortium. The core team worked with members of the MuSE Consortium to recruit 2–4 individuals from each of the above‐mentioned 10 groups of interest‐holders to co‐lead the development of the guidance. Co‐leads were selected for their expertise relevant to their interest‐holder group (e.g., lived experience of a health condition or research experience) and experience with health guidelines. We aimed to ensure co‐leads were balanced between high‐ and low‐ and middle‐income countries. In total, 26 co‐leads participated in decision‐making throughout critical stages of the guidance development [13]. The specific role of co‐leads in this study is described further under the section ‘data collection’.

The coordination of interviews was led by Olivia Magwood, a female doctoral candidate with previous experience with qualitative research projects and health guideline development. She worked with the co‐leads to develop interview guides, obtained institutional ethics approval, recruited participants, collected data through interviews, and led the analysis. Olivia Magwood had previously met some interview participants in a professional capacity (e.g., conference, workshop, guideline panel) but did not have any established relationships with them. Additional interviews were also conducted by a second female doctoral candidate (Lyubov Lytvtyn), a female research associate (Joanne Khabsa) and a male medical student (Alex Young‐Soo Lee). All additional interviewers were provided with orientation to the interview guides and consent forms prior to conducting interviews. This included reasons for doing the research and how the interview data would be used. None of the additional interviewers had relationships with the participants they interviewed.

### 2.2 Study Setting and Recruitment

All components of this pragmatic research study occurred virtually, allowing for the recruitment of participants from across the globe. We recruited individuals who self‐identified as belonging to any of the 10 groups of interest‐holders listed in Table 1. Participants were recruited through recommendations made by the MuSE Consortium network, snowball sampling, and social media. Recruitment occurred between May 2022 and November 2023. We prioritized recruitment of groups who co‐leads identified as playing the biggest roles in guideline development (e.g., patients, members of the public, providers of care, principal investigators).

**Table 1:** Interest‐holder groups to engage in guideline development [9].

Interest‐holder group	Description
Patients, patient caregivers, patient advocates/organizations	Those with lived experience with the condition of interest or who care for or advocate on behalf of those with lived experience
Payers of health research	Individuals and organizations that fund research projects, such as government funders, industry funders, foundations
Payers/Purchasers of Health Services	Individuals, organizations and entities that pay for health services
Peer Reviewed Journal Editors	Those who set journal policy on guidelines and manage the peer review process and editing
Policymakers	Individuals, organizations and entities that craft public or private policy (on health) at any level of government
Principal Investigators and all members of the of research team	Individuals, organizations, and associations that conduct or advocate health research
Product makers	Individuals working for companies that manufacture pharmaceuticals, medical devices, medical procedures, health technologies, or for profit educational and behavioral packages
Program managers	Managers/directors who plan, lead, oversee, or deliver any program that provides public health, community services, or clinical care (e.g., budgeting, hiring, staffing, organizing, coordinating, reporting)
Providers	Persons and their professional associations who provide health care in a professional capacity and allowed by regulatory bodies to provide a health care service
Public	Individuals in the general population of a defined geographic area excluding patients, caregivers, and health professionals living or working with the condition of interest

### 2.3 Data Collection

We co‐developed interview guides with the co‐leads from all MuSE interest‐holder groups (Appendix 1 presents an example interview guide, designed for interviews with patients, patient caregivers, and patient advocates/organizations). All co‐leads had the opportunity to develop questions specific to the key issues for their own interest‐holder group. The interview guides were developed iteratively over a series of meetings and email exchanges. Each interview guide included introductory questions to learn about the participant's role and experience with guideline development. Additionally, participants were asked about the GIN‐McMaster Guideline Development Checklist [12]. The checklist comprises 18 overarching topics. For each topic, respondents indicated whether or not they wanted to be engaged as decision‐makers or as advisors providing feedback, the results of which are described elsewhere [13]. Participants were also asked about conceptual principles and actionable strategies for working with them to develop health guidelines. Four team members (Olivia Magwood, Lyubov Lytvtyn, Joanne Khabsa, Alex Young‐Soo Lee) conducted interviews virtually using Zoom for a duration of 30–75 min. With participants' consent, the interviews were recorded and transcribed verbatim using an automated transcription app (Otter.ai). Transcripts were then imported into NVivo (version 14, Lumivero) for analysis. We anonymized all transcripts by assigning a unique identifier to each interviewee.

### 2.4 Data Analysis

We applied the principles of framework analysis to analyze the qualitative data from interviews. Framework analysis is a five stage process of familiarization with the data, identifying a thematic framework, indexing (applying the framework), charting and mapping, and interpretation [18]. The framework method is not aligned with a particular epistemological, philosophical, or theoretical approach, making it appealing for pragmatic research [19]. It can be adapted for use with deductive, inductive, or combined types of qualitative analysis. To guide the analysis, we selected the subcategories of principles and strategies reported by [20] as our preliminary deductive coding framework. This framework was developed through a review of reviews on key domains of research partnerships and identifies the principles, strategies, outcomes and impacts reported in different types of research partnership approaches. Principles and strategies were defined as “how research partnerships work” and included approaches, facilitators, characteristics and mechanisms of partnerships. The overarching principles and strategies identified by Hoekstra and colleagues are described in Appendix 2.

Two team members (Olivia Magwood & Caitlin Shyng) led the analysis. Both researchers re‐read the de‐identified transcripts and familiarized themselves with the data. All transcripts were imported into Nvivo for analysis. The two team members applied the framework and coded transcripts using the principles and strategies listed in Appendix 2 as the initial labels, identifying anything that might be relevant from as many different perspectives as possible. After coding the first few transcripts, the two authors met to discuss the labels they applied to ensure consistency in the coding approach before coding all remaining transcripts. One author (Olivia Magwood) compared the data coded by both authors and developed the initial themes and identified illustrative quotations. These themes were then reviewed by all members of the research team who conducted interviews (Lyubov Lytvtyn, Joanne Khabsa, Alex Young‐Soo Lee) and revised based on feedback and their reflections on the interviews they conducted. Additional illustrative quotations were selected to highlight the perspectives of program managers, principal investigators, and payers of health research. All team members reviewed the final findings and interpretations, drawing on our multidisciplinary expertise in engaged research.

### 2.5 Ethical Approval

This project received ethical approval from the Bruyère Health Research Institute (#M16‐21‐012), Ottawa, Canada.

## 3 Results

A total of 43 interest‐holders (65% female) from 15 different countries and all 10 interest‐holder groups participated in the interviews (Table 2). Interest‐holders had varied experiences in guideline development ‐ some participants had substantial experience in leading guidelines, many had participated as members of guideline development groups, and some were guideline naive but had an interest in engaged research approaches.

**Table 2:** Interview participant characteristics.

Interest‐holder group	No. interviews	Sex	Countries
Patients	7	5 Female 2 Male	Australia (*n* = 1), Canada (*n* = 2), UK (*n* = 3), USA (*n* = 1)
Payers of health research	1	1 Female	Canada (*n* = 1)
Payers of health services	1	1 Female	Argentina (*n* = 1)
Peer Review Editors	8	2 Female 6 Male	Argentina (*n* = 1) Australia (*n* = 1), Canada (*n* = 1), Columbia (*n* = 1), India (*n* = 2), UK (*n* = 2)
Policymakers	1	1 Female	Slovenia (*n* = 1)
Principal investigators	6	5 Female 1 Male	Brazil (*n* = 1), Canada (*n* = 1), Columbia (*n* = 1), Switzerland (*n* = 1), USA (*n* = 2)
Product makers	2	2 Female	Canada (*n* = 1), New Zealand (*n* = 1)
Program managers	3	2 Female 1 Male	Afghanistan (*n* = 1), Canada (*n* = 1), Estonia (*n* = 1)
Providers	9	4 Female 5 Male	Canada (*n* = 3), Croatia (*n* = 1), New Zealand (*n* = 1), Nigeria (*n* = 1), Peru (*n* = 1), USA (*n* = 2)
Public	5	5 Female	Australia (*n* = 1), Canada (*n* = 2), USA (*n* = 2)
**TOTAL**	**43**	**28 Female,** **15 Male**	**15 countries**

### 3.1 Principles

#### 3.1.1 Shifting from a Vertical Power Structure to a Horizontal Power Structure

Vertical and horizontal power structures differ in how power is distributed and decision‐making is organized. Vertical structures are hierarchical and centralized, while horizontal structures are more egalitarian. Traditionally, principal investigators and health care providers held positions of power throughout guideline development, leading the process and making the final decisions regarding health recommendations based on “the narrative of the clinical assessment of need”. Communication and information tend to flow from the top down, and feedback from others may be limited. Engaging diverse interest‐holders demands a paradigm shift to a horizontal power structure, where power is distributed more evenly across individuals or groups, rather than being concentrated at the top of a power hierarchy. This structure emphasizes collaboration, equality, and shared decision‐making throughout the guideline development process.

The root of all of this is a hierarchy, like a vertical power structure versus a horizontal power structure […] Ideally, these [patients and healthcare providers] should be on an equal footing, my voice as a patient should have equal value to the healthcare professionals voice. Unfortunately, we still have some way to go to establish that… a lot of what we're talking about today is underpinned by power.

Patient 2, male, UK

Several patients and members of the public emphasized the importance of being treated as a peer alongside healthcare providers and principal investigators and being provided with opportunities to make decisions together.

There isn't anything important that researchers should decide by themselves, any more than there's anything important about research, that patients should be deciding by themselves. We must start making these decisions together.

Patient 5, female, UK

Providers agreed with this sentiment, emphasizing the importance of “some sort of paradigm shift to […] shared decision making” (Provider 2, male, Nigeria). Members of the public highlighted that this shift in power towards shared decision making needs to be based on relationships built on trust, respect and “the belief of those people at the table [providers and principal investigators] that I should be there and I can contribute” (Public 3, female, Canada).

#### 3.1.2 Promotion of Epistemic Justice

The term “epistemic justice” describes fairness and equality in the production, dissemination, and validation of knowledge [21]. Epistemic justice emphasizes the recognition of diverse perspectives, experiences, and forms of knowledge, particularly those that have historically been marginalized or excluded. Co‐production of knowledge was consistently described as an essential principle of guideline development to ensure that guideline recommendations address patient needs. One patient explained that there needs to be epistemic justice for patients and members of the public to share knowledge production spaces, “somewhere where we meet to establish common understandings, common narrative, shared learning” alongside clinicians in order to provide a complete knowledge base to inform guideline development.

Knowledge doesn't belong to anyone…any one group who thinks they've got all the knowledge they need is very delusional. […] like a three legged stool, you can't have a two legged stool, you fall off. And if your two legs are academic knowledge and clinical knowledge, you're gonna keep falling off. So what you need is the knowledge of lived experience to stabilize you, then you've got a complete knowledge base. And that stands a chance of creating an evidence base that will lead to better care that will actually address all the needs of the clinicians and critically, the patients and people who care for them.

Patient 5, female, UK

Applying an epistemic justice approach to guideline development requires “embracing the not knowing” (Patient 5, female, UK) and encouraging bi‐directional conversations that promote co‐learning, where “we are going to learn from them [patients] as much as we're teaching them…maybe we'll learn from them more” (Principal investigator 4, female, USA). Importantly, one member of the public highlighted that patient experience is often viewed as “anecdotes and stories” (Public 2, female, Canada), and it does not receive the same weight and value as quantitative research. In order to embody epistemic justice in guideline development, knowledge based on experience needs to be valued as highly as traditional academic knowledge:

If you've got patients who've got very little kind of educational or so‐called ‘academic ability’, but we've got extremely important lived experience that needs to be conveyed, then you need to find those places for them in the research world where they can provide that knowledge.

Patient 5, female, UK

#### 3.1.3 Representation

Interest‐holders generally recognized the importance of ensuring that diverse perspectives were represented within a guideline development group. However, how “representation” was described differed. For some interest‐holders, representation meant that different groups, such as patients and providers of health care, were involved in decision making. For others, representation meant ensuring that geographic representation within a specific discipline was considered so that guideline recommendations would have local buy‐in across a country. One provider of care emphasized the importance of including providers of care across regional health authorities to ensure uptake and paying special attention to Indigenous representation in their country context (Provider 8, male, New Zealand). However, a program manager explained that they have moved away from a specialty‐based model, which focused on engaging people from specific clinical areas, towards an expertise‐based model which could span several specialty areas:

So we've moved away from a specialty based model… we don't say ‘you must be a college holding pediatric infectious disease specialist’, we say the committee has to cover off these areas of expertise […] it might be that we have a PhD epidemiologist who also is an infectious disease doctor, so they actually are satisfying the requirement to have expertise in multiple areas […] that is outlined in the terms of reference, like it describes the content areas that are expected to be represented.

Program manager 2, male, Canada

Patient representation was discussed most in depth, primarily around concerns as to the ability of patients to represent the wider patient population:

But when is the person speaking from their own lived experience? And when are they claiming, rightly or wrongly, to represent cohorts of patients? I think a lot of people call themselves a representative, but then they speak from their own personal lived experience and are not really representing. Whereas when I claim to represent, that's because I've gone into my networks, and I've consulted hundreds of people […] often I'll end up in a conversation with 100 or so people back and forth, back and forth, I say, what questions do you want me to ask? What information do you want me to pass on? And they'll tell me.

Patient 3, female, Australia

Additionally, principal investigators commented on the pragmatic decisions that guideline developers need to make, recognizing that it is not possible to have every patient, provider or program manager perspective represented in a single guideline project. They emphasized that, rather than “checking a box” by including individuals from several interest‐holder groups, guideline developers should focus on including the perspectives that are essential for making the relevant guideline decisions, such as a patient “who had the major side effect from a certain intervention, or […] who's not insured” (Principal investigator 4, female, USA)

#### 3.1.4 Avoiding Tokenism and Promoting Diversity

When engagement in guideline development is done tokenistically, a small number of individuals from underrepresented groups may be involved to give the appearance of diversity or inclusivity. Their presence may serve to deflect criticism or fulfill diversity quotas without meaningfully influencing the guideline initiative with their perspectives or experiences. Despite their inclusion, token individuals may have limited influence or decision‐making power within guideline development. They may be excluded from important discussions or marginalized in decision‐making processes.

When you have an unknown health condition and you have a PhD kind of linked to health research, you get wheeled out and asked to participate in certain things for representation, I guess.

Patient 2, male, UK

It's just that the way they went about it, they didn't bring in the lived experience people and the patient organization until after these academics had created the draft.

Patient 3, female, Australia

I feel that I live in a PPI [patient and public involvement] cupboard. Every now and again, somebody needs some PPI. So they open the cupboard door, pull me and a few others out, sit us down and say this is what we want. This is what we're going to do. Is that alright with you? Okay, back in the cupboard, and we'll see you another time.

Patient 5, female, UK

Overall, tokenism undermines genuine efforts to promote diversity, equity, and inclusion by reducing engagement to mere symbolic gestures rather than addressing systemic issues of exclusion. Patients recognized how this might exacerbate ongoing issues related to equity and diversity in guideline development. One patient (Patient 4, female, Canada) described most patient advocates as “a 50‐something white woman” and explained that guideline developers need to work harder to engage young parents, older people, new immigrants and Indigenous people. This was echoed by a second patient, who also mentioned that patient representatives are generally “white people that speak English and that have a high degree of education” (Patient 6, female, Canada).

### 3.2 Strategies

#### 3.2.1 Governance and establishing committees

Patients recommended that a key strategy in ensuring their meaningful engagement in guideline development was involving them in governance alongside providers of care. Governance of guideline development includes overseeing the organization, budget and planning of the initiative. It also involves establishing clear roles, responsibilities, and reporting structures for project team members. Several patients believed that they should be involved in the governance of health guideline development.

I think that they should be involved in governance. Providers and patients and caregivers have the capacity and the skill and the relationships to be involved in making decisions from the beginning.

Patient 1, male, USA

Another patient described that the “right” people for this role should have “an appetite for change, who've got vision, who've got […] the kind of emotional energy that's needed to bring others along with them.” (Patient 5, female, UK). It was recognized, however, that having experience of a health condition would not be sufficient to fill this role ‐ patients would also need to have strong language and scientific skills. Recruitment for these roles could be done through patient organizations and their social media channels, which are well connected to a broad range of patients in various locations.

If it is not possible to be involved in governance, one principal investigator (Principal investigator 6, female, Canada) suggested that some interest‐holder groups including patients, policymakers and program managers could be involved in other committees or working groups focused on specific areas of the guideline to allow them to engage in defined activities during guideline development. This includes acting as guideline development panel members, external review group members, and peer review group members. Interest‐holders could also be engaged externally to the guideline development group, for example, as survey participants. It was noted that program managers, particularly those who may be too busy to engage regularly throughout the process, could be invited to participate in workshops at the end of the initiative:

If someone was at the provincial level, and it was not possible for them to be part of that [working group]. but it was very important to be engaged or provide inputs…. In this case, we just say to them, ‘Okay, when we complete the first draft, we will share with you that document and then we'll have your point of view’ or also, […] when we complete it, in order to have a wide range of engagement, we organize workshops at the national level, and invite all the key stakeholders to be part of that and having their inputs on that.

Program manager 1, female, Afghanistan

In cases where there are insufficient resources to establish these committees or additional engagement activities, it was also recommended to look for published literature on interest‐holder priorities:

I even say to a group, if you don't have the capacity to be having these extra committees and doing these large surveys and everything, then just look to the literature to what's already been done about stakeholder views, etc. That's something relatively easy without actually trying to find people and involve them and bring them in and have meetings with them. … And, you know, they think, Oh, well, that's not really involving them. But it is, you're still getting information about their priorities.

Principal investigator 6, female, Canada

#### 3.2.2 Setting Clear Expectations and Timelines

Clear expectations ensure that everyone understands their roles and responsibilities within a guideline development initiative. One peer‐review journal editor described this as “setting the boundaries and outlining your ask” (Peer reviewed journal editor 8, male, UK). When roles and timelines are well‐defined, individuals can focus their efforts on tasks that directly contribute to achieving objectives. Clear expectations contribute to interest‐holder satisfaction by ensuring that deliverables are produced on time and meet the agreed‐upon standards. This enhances trust and credibility with interest‐holders, which is essential for maintaining positive relationships. When inviting interest‐holders to join a guideline initiative, guideline developers should include:

a description of what's expected, how much of my time it is going to take, what is expected of me. So I always asked that question, what are you expecting? Are you expecting me to be writing these portions of the grant? Or are you expecting me to feed back some comments? […] I think, if you understand clearly that there's this many meetings, like monthly meetings or quarterly meetings, and it's going to be a full day or it's 2 hours […] those expectations should be clearly laid out before somebody gets involved.

Patient 6, female, Canada

Additionally, patients in particular expressed frustration with unclear or unrealistic timelines. This included timelines which were too short to adequately review documents and provide meaningful feedback, but also irritation with the long process of guideline development. One patient described that, while she had energy several years prior, the slow pace of academic work and her declining health meant that she had less energy to stay committed with projects: “for people who like a deadline, like, it's really slow” (Patient 4, female, Canada). This was echoed by a second patient:

I think it's even if the goodwill is there, and they could have been at the beginning of a year, the second year, their conditions deteriorate and they can't do it any longer. So I think those are sort of inequities that have got to be thought about and addressed.

Patient 7, female, UK

To mitigate this frustration, regular communication should be communicated to all interest‐holders engaged in the guideline development project. One provider expressed the importance of “having clear agendas, having clear, regular meetings, making sure that the agendas are followed, and that decisions and actions are documented. Clearly, all of that kind of information, or all of that planning is really, really vital to success” (Provider 7, female, Canada).

#### 3.2.3 Compensation and Acknowledgement

Patient and public interest‐holders may have different motivations and expectations regarding compensation compared to other interest‐holders who engage in guideline development. For other interest‐holders, participation in research or guideline activities is often an implied part of their job that helps to advance their career. In contrast, patients and members of the public often take time off work, caregiving duties or other regular daily activities in order to contribute. Patients and members of the public expressed mixed feelings regarding compensation. Some patients reported turning down all compensation, others expressed that patients should be compensated for their out‐of‐pocket expenses but not for sharing their experiential knowledge, and others felt that offering compensation for the time spent towards a guideline was a good way to recognize patients' contributions and value. These differences in opinion may have been related to country context. For example, one Canadian patient explained that accepting financial compensation could influence a person's eligibility to receive the Canada Pension Plan disability benefit. In other contexts, such as the UK and Australia, patients explained that including budget items in grant applications to support consultation and co‐design with patients was an expectation and improved chances of receiving funding. Engaging patients early in grant submissions was suggested as a way to plan for these costs:

I get really cross when in the budget and the planning and the organizing there are false assumptions made about what will be accessible and inclusive engagement with consumers, and what the costs and what the resources will be that are required to make it accessible and inclusive. So to me, I would want to be involved [early], or know that there's someone involved who understands our patients.

Patient 1, male, USA

Another patient suggested that organizations or institutions who develop guidelines should have an organizational‐level group or committee to assist in the pre‐application grant process. This group could review grant submissions and indicate strategies to improve patient and public engagement, including providing suggestions for compensation. Patients also suggested that, in situations where financial compensation cannot be accepted, co‐authorship on guideline documents could be a potential mechanism to recognize patient and public contributions. One patient shared an example of poor acknowledgement of their academic contributions:

It's actually quite terrible. They put out a paper and they didn't even put a thank you to the patients […] I know some patients who'd spent hours looking through all the research questions that had been submitted. They didn't even get the acknowledgement, let alone co‐authorship, and they really should have been co author, and therefore as co author, they should have maybe seen the piece before it was published… a very bad example.

Patient 4, female, Canada

#### 3.2.4 Strategies to Facilitate Engagement Throughout the Guideline Development Process

Across all interest‐holder groups, there was a clear preference to be engaged early in the guideline development initiative, particularly in setting priorities, topic selection and research question generation. Engagement in other aspects of guideline development was more variable depending on the technical skills required. Some strategies were identified to support interest‐holder engagement in technical processes such as evidence reviews and developing recommendations:

1. *Conduct interest‐holder mapping:* Program managers highlighted the importance of mapping exercises to identify and recruit the key players working in the field of the guideline. They emphasized that the individuals with the highest level of interest should be engaged to ensure retention throughout the guideline development project.
2. *Mentorship*: For patients and members of the public, a mentorship program was suggested whereby less experienced patients/public could “buddy up” with a more experienced patient/public member to guide them through the guideline development process to help their advocacy efforts be most effective.
3. *Facilitation by a methodologist or chair:* One principal investigator recognized that some interest‐holders, such as program managers, may not be familiar with technical processes such as determining the strength of the evidence. They suggested that guideline group discussion includes a skilled methodologist or chair who could facilitate discussions about interpretation, such as the size of the benefits and harms.
4. *Leveraging technology and online meeting spaces:* Patients were generally supportive of online engagement opportunities because it allows patients from different geographic locations to participate in guideline development. However, one principal investigator cautioned that online meetings are only effective for small group discussions. When larger groups need to meet, it can be difficult to ensure that everyone gets a chance to provide their input in an online environment. While online meetings can help reduce guideline development costs, it may still be necessary to invest in in‐person meetings and travel expenses. They highlighted that online tools, such as GRADE Panel Voice, can help coordinate the voting processes.
5. *Meeting people “where they are, as opposed to asking them to come to you”*: One patient described a successful approach to engaging with other patients is meeting in familiar local spaces within the community rather than academic locations such as a university. They described the success of meeting with patients at the local pub, knowing that the patients lived nearby, parking was accessible, the food was enjoyable, and the patients knew where to locate an accessible washroom.

#### 3.2.5 Expanding Opportunities for Engagement in Dissemination Activities

Several interest‐holders emphasized the importance of looking beyond academic publications as an opportunity for dissemination. Interest‐holders should be empowered to plan and participate in dissemination activities:

People can also be empowered to present about this. So a lot of the time again, you see that only the chair has one session in one meeting, they present about a certain guideline, which is completely the wrong approach. You actually want to see that every person on that panel have an opportunity to showcase the work, because then they feel it's their responsibility to do this dissemination […] it makes people feel like it's not ‘I'm just there and I'll get a publication out of it’. It's way bigger and more important.

Principal investigator 4, female, USA

For some interest‐holder groups, this may mean presenting at annual academic meetings that are specific to a clinical or public health specialty. However, other strategies were also suggested, such as developing online education sessions led by providers, or developing social media campaigns led by patients, caregivers and members of the public. Planning for these dissemination activities should occur early and be re‐visited regularly during the guideline development process. One payer of health research explained that maintaining close relationships with implementing organizations can facilitate dissemination activities:

And so then we already have that sort of dissemination implementation mechanism […] because we have that standing, ongoing connection with the provinces and territories. Other than that, as soon as we release new guidance, we have a whole sort of media stream that we work with, to get the information out there just to the general public.

Payer of health research 1, female, Canada

#### 3.2.6 Consider When Engagement May Not Be Feasible

While most interest‐holders recognized the importance of engagement in guideline development, they also highlighted several circumstances where engagement might be limited or not be possible. For example, in emergency contexts where rapid guidance is required, such as during the COVID‐19 pandemic, it was recognized that only a subset of interest‐holders (such as providers of care) would likely be engaged, and that guideline developers would need to make pragmatic decisions about the most essential interest‐holder groups to engage in decision‐making.

Additionally, two providers of care discussed that some countries, such as low‐and‐middle income countries or smaller countries, may not have capacity for full de novo guideline development, but rather may rely on guideline adaptation (such as GRADE‐Adolopment [22];) to inform their own national guidelines because they lack “*the sort of logistics in terms of funding, and […] the appropriate capacity to sit in the panels*” (Provider 2, male, Nigeria). However, they did describe that it could be possible to engage with interest‐holders who may not have been engaged in the original guidelines as part of the adaptation process to enrich the perspectives considered within their own national guideline documents.

## 4 Discussion

The findings of this paper complement ongoing work by the MuSE Consortium to develop guidance for the engagement of interest‐holders in guideline development [13]. Through interviews, we identified several principles and strategies to promote engagement across 10 different groups of interest‐holders. The principles we identified were establishing horizontal power structures, promoting epistemic justice and focusing on co‐production of knowledge, conceptualizing “representation” both across and within interest‐holder groups, as well as avoiding tokenism and promoting diversity. Effective strategies for meaningful engagement included involving interest‐holders in governance, setting clear expectations and timelines, providing compensation and acknowledgment for contributions, and providing opportunities for engagement in dissemination activities. We also identified specific strategies to facilitate engagement throughout the guideline development process. In addition, we highlight instances where engagement may not be feasible, including in emergencies where rapid guidance is required.

The findings of this research are a major strength in that the principles and strategies for engagement come directly from interest‐holders themselves. Previous research in the area of principles and strategies for engaged guideline development has relied on published literature (e.g., [15, 16]; Magwood et al. revision submitted) and was lacking in evidence across many interest‐holder groups. However, some interest‐holder groups (such as patients, public, providers of care, and principal investigators) are oversampled among our interview participants. While these may represent the most commonly engaged groups in guideline development [15], this also means that our knowledge of the principles and strategies of some under‐sampled groups of interest‐holders remain limited, as we did not achieve data saturation for each interest‐holder group. Despite this, many principles and strategies identified in this study were consistent across interest‐holder participants, suggesting that there are some core principles which apply to guideline development regardless of interest‐holder group. In addition, we believe that many of the identified principles and strategies also apply outside the context of guideline development (e.g., in health research more generally).

Representation remains a key issue for interest‐holder engagement in the development of guidelines [9], and this was echoed by interview participants. Current guidance on identifying interest‐holders from the MuSE Consortium highlights that engaged individuals should be able and willing to represent an interest‐holder group, but does not provide concrete steps on how to do so [23]. Smith et al. [24] proposed a representation framework to serve as a guide for recruitment of interest‐holders which incorporates four elements: ‘nominated membership’ (members who are nominated by their organizations), ‘dispositional representation’ (individuals are engaged by virtue of their job role), representation of ‘shared interest’ (advocates speaking on behalf of a group) and ‘personal representation’ (individuals with personal experience of the condition of interest). However, they also highlighted a pragmatic consideration for interest‐holder selection: how informed or skilled interest‐holders need to be in relation to the task at hand [24]. Indeed, it is unlikely that the average patient or healthcare provider, for example, has the necessary interest and experience to fully engage in health guideline development. Further research on how to recruit interest‐holders and balance their representativeness and expertise within the context of guideline development is warranted.

Although principles are not “how to” guides, they should clearly identify what is desirable and ideal and be linked to action so that guidance can be developed based on those foundational principles [16]. The current study informs the MuSE Consortium's guidance for engagement in guideline development [13]. These findings also complement other guidance available from the MuSE Consortium to guide engaged research approaches, including practical guidance for involving interest‐holders in health research [25], factors to consider during identification and invitation of individuals in a research partnership [23], and a new taxonomy for defining conflicting and non‐conflicting (legitimate) interests of interest‐holders in health research [26]. The MuSE Consortium has also begun a project to further explore the engagement of interest‐holders in evidence synthesis [27].

### 4.1 Strengths and Limitations

While we interviewed ten different groups of interest‐holders, recruitment across groups was unbalanced. We experienced challenges in recruiting several groups including program managers, policymakers, payers of health research and health services, and product makers. This may be reflective of the disproportionate membership of the MuSE Consortium itself (which has, to date, mostly attracted principal investigators, providers of care, and patients/public members). This may also reflect these groups (un)willingness to participate in interview‐based research or limited experience in guideline development. Our project was also constrained by its timing during the COVID‐19 pandemic, when many program managers or policymakers may have had competing priorities (e.g., developing rapid guidance for COVID‐19, vaccination implementation, etc). Additionally, while we aimed to recruit interviewees globally, participants (particularly patients and members of the public) were predominantly from high‐income English‐speaking countries. This generally reflects current practices in engaged health research [28], and more effort is needed to offer opportunities and build capacity in low‐ and middle‐income countries.

## 5 Conclusions

Engagement is time consuming and resource intensive and requires an important shift in power structures and governance. Guideline developers should do their best to integrate interest‐holders in committees, engage them as early as possible in the process, provide them with rightful acknowledgment, be clear about expectations and timelines, and facilitate their engagement throughout the guideline development process. Guideline developers may need to make pragmatic decisions as to who they engage in guideline development and how, including at which stage of the process, and with what decision‐making power. Constraints may exist when rapid guidance is needed or in settings with poor capacity for evidence synthesis and guideline development. Capacity strengthening in low‐ and middle‐income countries may help address current disparities in engagement in guideline development.

Future research should explore issues around representativeness of interest‐holders and testing of our proposed strategies. Additional investigation on the perspectives of program managers, policymakers, and payers of health research and health services would enhance the current findings.

## Author Contributions

Conceptualization: Olivia Magwood, Peter Tugwell, Kevin Pottie, Jennifer Petkovic. Methodology: Olivia Magwood, Jennifer Petkovic, Peter Tugwell, Kevin Pottie, Vivian Welch. Data Curation: Olivia Magwood, Lyubov Lytvyn, Joanne Khabsa, Alex Young‐Soo Lee. Formal analysis: Olivia Magwood, Caitlin Shyng. Writing ‐ Original Draft: Olivia Magwood. Writing ‐ Review and Editing: All authors. Supervision: Peter Tugwell, Kevin Pottie. Project administration: Olivia Magwood. Funding acquisition: Peter Tugwell, Vivian Welch, Jennifer Petkovic, Kevin Pottie.

## Conflicts of Interest

The authors declare no conflicts of interest.

## Data Availability

The data that support the findings of this study are available from the corresponding author upon reasonable request.

## Appendix 1 INTERVIEW GUIDE (Patients)

**Section 1: Getting to know you**

First, I'd like to know a little about you. Could you please tell me about yourself?

*e.g., where do you live, do you work, do you also have other roles (e.g., researcher, editor, program manager) etc*.

Thank you. Next, we would like to get your opinion about how and when interest‐holders should be engaged in the guideline development process.

When we say ‘guidelines’ we are referring to clinical practice guidelines, which guide decision making about treatments, as well as public health guidelines, such as those you're probably familiar with related to COVID‐19, like wearing a mask when in public.

When thinking about guidelines, which term do you prefer to describe yourself:

1. Patient

2. Consumer

3. Member of the public

4. Other?

Are there differences between these terms? If yes, what are they?

Could you please tell me about your experience in health guideline development?

- *Have you ever been involved in a guideline panel?*
- *How many years of experience do you have in this area?/How many guidelines?*
- *Do you remember the type of guideline you were a part of?*
- *What was your role in the guideline development process?*

**Section 2: GIN‐McMaster checklist**

Last year we surveyed interest‐holders from around the world. They gave us their opinions on when they should be engaged in guideline development. At each stage, they had the choice of selecting “engagement not required”, advice/feedback, or decision‐making.

Advice/feedback means that the stakeholder gives their opinions, and the guideline development team gets to decide whether they want to use those opinions.

Having a decision‐making role means that stakeholder want an equal “vote at the table” – the guideline team *must* take their vote under consideration.

Now, I would like to show you the 18 stages of the GIN‐McMaster checklist that patients have provided us feedback on how they want to be involved.

In the table on the screen, you can see that there are some stages where we have heard consistent opinions about engagement. In other stages, we are still unclear/unsure about how patients/public want to be engaged

We want to know

1) Do you agree with level of engagement indicated at each stage?

2) What are your opinions about the stages marked as “unclear”?

**For Patients/Caregivers:**

**Table:**

GIN‐MCMASTER CHECKLIST	Engagement
1. Organisation, budget, planning	unclear
2. Priority setting	Decision‐making
3. Guideline group membership	Decision‐making
4. Guideline group processes	Decision‐making
5. Identifying target audience and topic selection	unclear
6. Consumer and stakeholder involvement	Decision‐making
7. Conflict of interest considerations	unclear
8. (PICO) Question generation	Decision‐making
9. Considering importance of outcomes and interventions, values, preferences and utilities	Decision‐making
10. Deciding what evidence to include and searching for evidence	unclear
11. Summarizing evidence and considering additional information	unclear
12. Judging quality, strength or certainty of a body of evidence	unclear
13. Developing recommendations and determining their strength	unclear
14. Wording of recommendations and of considerations of implementation, feasibility and equity	Decision‐making
15. Reporting and peer review	Decision‐making
16. Dissemination and implementation	Decision‐making
17. Evaluation and use	unclear
18. Updating	unclear

**Section 3: Scenario**

Imagine that a new clinical practice guideline is being development for the **treatment of COVID‐19 (e.g., a new drug).** How and when do you think patients/caregivers/public should be engaged?

**Section 4: Final remarks**

- If you could give one piece of advice to a guideline development team on how to engage with patients/public, what advice would you give to them?
- Please tell me anything you think we should know about engagement in guideline development.
- Is there anything else you would like to share?
- Do you have any questions for me?

Thank you [end interview].

## Appendix 2 Principles and strategies of research partnerships [20]

**Table:**

Subcategory	Description
**Principles**	
Relationship between researchers and stakeholders	Partners build and maintain relationships based on trust, credibility, respect, dignity, and transparency Partners acknowledge, reward and value the diverse expertise of the partnership and its members Partners share in decision‐making and leadership of different research activities The partnership addresses power dynamics within the team and aim to promote equity, self‐determination and/or social justice The partnership ensures representation and/or inclusivity and addresses disciplinary and sectoral issues
Co‐production of knowledge	Partners co‐produce knowledge and meaningfully engage stakeholders at different phases of the research process Partners ensure that all members of the partnership have ownership over the data and resulting knowledge products Partners strive to balance the need for scientific rigour alongside the practical need for actionable knowledge Partners ensure the long‐term implementation of the findings in real world settings and systems
Meaningful stakeholder engagement	Partners carefully plan and regularly reflect on their strategic approach to collaboration Partners are flexible and creative in the collaborative research activities and tailor the approach Researchers and stakeholders benefit from the partnership The partnership identifies the stakeholder's needs and makes sure that the research is relevant for the stakeholders
Capacity‐building, support and resources	Partners build capacity among all members of the partnership Partners ensure bidirectional exchange of skills, knowledge and capacity between members of the partnership
Communication between researchers and stakeholders	The partnership fosters regular, open, clear and honest communication between its members
Ethical issues of collaborative research activities	Ethical issues of collaborative research activities
**Strategies**	
Relationship between researchers and stakeholders	Initiate partnership and identify the team members; the partnership can be initiated by researchers or stakeholders; researchers can use targeted or open strategies to identify the stakeholders Monitor, experiment with and evaluate the collaborative research activities on an ongoing basis Work together to develop and define norms, rules and expectations in terms of timelines and tasks; this includes defining the level of stakeholders’ engagement, roles and commitment Use a variety of activities to foster collaboration, communication and respect amongst the team members; strategies can include, but are not limited to, creating a common language, negotiating and addressing conflict, tailoring meets to the needs of the team, and providing opportunities to socialise
Capacity‐building, support and resources	Provide opportunities to educate and train all team members; this strategy may include training that supports capacity for collaboration or research methods Provide time, resources and funding to support the collaborative research activities; stakeholders may be paid for engagement in the research process Provide practical and emotional support to stakeholders to help overcome barriers to engagement
Communication between researchers and stakeholders	Use a variety of methods to facilitate communication amongst team members; strategies include, but are not limited to, verbal methods (e.g., structured meetings, brainstorm sessions), written methods (e.g., email discussions, surveys) and visual methods (e.g., photovoice); this communication can be done in‐person or via mediated methods (e.g., teleconference, online)
Stakeholder engagement in the planning of the research	Strategies include, but are not limited to, stakeholder engagement in identifying or refining the ‘research questions’, stakeholder engagement in development the ‘research protocol’, stakeholder engagement developing or refining ‘research instruments’ (e.g., questionnaires, interview guides) and stakeholder engagement in development of participant ‘information material’ (e.g., informed consent)
Stakeholder engagement in conducting the research	Strategies include, but are not limited to, stakeholder engagement in ‘data collection’ (e.g., recruitment of participants, study outcomes, conducting interviews, conducting literature review), stakeholder engagement in data analysis, and interpretation of findings
Stakeholder engagement in dissemination and application of the research	Strategies include, but are not limited to, stakeholder engagement in ‘writing reports or scientific papers’ (e.g., stakeholder is co‐author on a scientific paper), stakeholder engagement in ‘presenting findings’ to academic and community audiences, stakeholder engagement in a ‘developing and implementation action plan’ to ensure findings are used, and stakeholders use the findings to create change
